# Association between maternal age and sex-based neonatal free triiodothyronine levels

**DOI:** 10.1186/s12902-024-01631-3

**Published:** 2024-06-27

**Authors:** Yanmin Chen, Tao Shen, Yuhua He, Xinning Chen, Danqing Chen

**Affiliations:** 1 Obstetrical Department, Women’s Hospital School of Medicine Zhejiang University, Hangzhou City, Zhejiang Province 310006 China; 2 Clinical Trial Ward, Women’s Hospital School of Medicine Zhejiang University, Hangzhou City, Zhejiang Province 310006 China; 3 Department of Obstetrics and Gynecology, Shanghai Jinshan Tinglin Hospital, Shanghai City, 200500 China

**Keywords:** Maternal age, Thyroid hormones, Triiodothyronine

## Abstract

**Background:**

Advanced maternal age may affect the intrauterine environment and increase the risk of neurodevelopmental disorders in offspring. Thyroid hormones are critical for fetal neurological development but whether maternal age influences fetal thyroid hormone levels in euthyroid mothers is unknown.

**Objective:**

This study evaluated the association between cord blood thyroid hormones and maternal age, fetal sex, maternal thyroid function, and other perinatal factors.

**Methods:**

The study population consisted of 203 healthy women with term singleton pregnancies who underwent elective cesarean section. Maternal levels of free T3 (fT3), free T4 (fT4) and TSH before delivery, and cord levels of fT3, fT4 and TSH were measured. Spearman’s correlation coefficient and multiple linear regression analyses were performed to determine the correlation between cord thyroid hormone parameters and maternal characteristics.

**Results:**

There were no significant differences in maternal serum or cord blood thyroid hormone levels between male and female births. In multivariate linear regression analysis, maternal age and maternal TSH values were negatively associated with the cord blood levels of fT3 in all births, after adjusting for confounding factors. Maternal age was more closely associated with the cord blood levels of fT3 in female than in male births.

**Conclusion:**

The inverse association between maternal age and cord blood levels of fT3 in euthyroid pregnant women suggested an impact of maternal aging on offspring thyroid function.

## Background

Maternal age has increased in the last decades in many developed countries. A similar trend has occurred in China, with the implementation of universal two-child policies and socioeconomic development. Advanced maternal age is defined as ≥ 35 years at the time of childbirth and it is strongly associated with various adverse pregnancy outcomes, such as fetal growth restriction, intrauterine fetal death, and birth defects. Epidemiological studies have shown that maternal age is an independent risk factor for neurodevelopmental disorders, including autism spectrum disorders [1–3] and attention-deficit/hyperactivity disorder [3, 4]. Data from animal studies also relate advanced maternal age to the impaired cognitive and emotional development of offspring [5, 6]. Otherwise, advanced maternal age may increase the risk of cardiovascular disease in offspring during their adulthood [7]. The molecular mechanism underlying the various health risks subsequently faced by the offspring of older mothers has not been fully elucidated. Some studies have suggested that impaired uteroplacental structure and function in older mothers result in reductions in fetal blood volume and nutrient transfer, which may adversely affect offspring health [8–10].

Thyroid hormones are critical for fetal and neonatal development before and after birth. Extensive research has shown that maternal thyroid dysfunction is associated with an increased risk of neurological and psychiatric disorders in offspring. Thyroid hormone transfer from mother to fetus persists throughout gestation, even if the fetus is capable of producing its own thyroid hormone. Cord blood thyroid hormone levels can reflect fetal thyroid hormone status and may influence fetal growth *in utero* [11]. Furthermore, changes in thyroid hormone levels in the cord blood of euthyroid mothers are associated with altered neurodevelopment during infancy and childhood [12, 13]. Changes in the placental expression of deiodinase and decreased levels of free T4 and total T3 in the umbilical cord blood of women with gestational diabetes have been reported [14]. While advanced maternal age is associated with placental and utero-placental dysfunction, fetal thyroid hormone levels in advanced age mothers with normal thyroid function have not been adequately investigated. In this study, thyroid hormone levels in the cord blood of euthyroid mothers were assessed with respect to their possible association with maternal age, thyroid function and other perinatal factors.

## Methods

### Participants

A cross-sectional study was conducted at Women’s Hospital Schools of Medicine Zhejiang University. Singleton pregnant women who underwent scheduled cesarean section were enrolled from March to June 2018. Indications for cesarean delivery included scarred uterus, breech position, macrosomia, and cephalopelvic disproportion. Exclusion criteria included women with pregnancy complications (e.g., gestational diabetes, pregnancy-induced hypertensive disorders, intrahepatic cholestasis of pregnancy); a history of thyroid disorders or those treated with levothyronxine during pregnancy; hypertensive disorders, renal diseases, diabetes, or other serious medical conditions; who conceived by assisted reproductive technology; preterm delivery; and with incomplete medical record data. The study was approved by the Institutional Ethics Committee of the hospital (IRB-20,230,307-R). Written informed consent was obtained from all participants.

Maternal serum thyroid hormones are routinely screened at hospital admission for delivery. Cord blood was sampled from the umbilical vein after delivery. Free T3, free T4 and TSH levels of maternal and cord blood serum were measured via chemiluminescent microparticle immunoassays using the ARCHITECT i2000SR (Abbott Laboratories, Abbott Park, IL, United States) at the clinical laboratory of Women’s Hospital School of Medicine Zhejiang University. All reagents were obtained from Abbott and used as per the manufacturer’s instructions.

### Statistical analysis

Categorical variables are reported as the frequency and percentage, and continuous variables as the mean ± SD or median (interquartile range). The distributions of continuous variables were checked using a histogram and a Kolmogorov-Smirnov test. Normally distributed continuous variables were compared using independent sample t-tests, and variables not normally distributed were compared using Mann-Whitney U tests. Spearman’s coefficients (r_s_) were calculated in bivariate correlation analyses exploring the relationships among continuous variables. Multiple linear regression models were used to analyze the relationship between maternal age and the cord blood levels of fT3, controlling for the effects of maternal body mass index (BMI), thyroid-stimulating hormone (TSH) level and birthweight. Residual plots showed that the residuals were randomly distributed around 0, suggesting that the multiple linear regression models were appropriate. P–P plots also showed that the residuals were normally distributed.

All regression tests were performed using the Enter method. A P value < 0.05 was considered to indicate statistical significance. Statistical analyses were performed using SPSS software (version 26.0). The correlation matrix was visualized using Origin 2022 software (OriginLab corporation, USA) and the “Correlation Plot” package.

## Results

Two hundred and three pregnant women were ultimately included in the analysis. Their baseline characteristics are presented in Table 1. There were no significant differences in maternal characteristics with respect to male vs. female births, including maternal age, parity, gestational age, and BMI at delivery. Average birthweight was significantly lower in female than in male births (3432 ± 387 vs. 3489 ± 417, *P* = 0.024). Differences in maternal serum and cord blood thyroid hormones associated with neonatal sex were also not significant.

**Table 1:** Study population characteristics (*N* = 203)

Characteristics	All births (*N* = 203)	Male births (*N* = 108)	Female births (*N* = 95)	*P* value
*Mother*				
Age (year)	34.11 ± 4.15	33.67 ± 4.06	34.61 ± 4.21	0.106
Primiparous	37 (18.2%)	21 (19.4%)	16 (16.8%)	0.632
Gestational Age (wk)	38 (38, 39)	38 (38, 39)	38 (38, 39)	0.114
Pre-pregnancy BMI (kg/m^2)^	20.96 (19.43, 23.12)	21.14 (19.40, 23.14)	20.90 (19.43, 23.05)	0.970
BMI (kg/m^2)^	26.97 ± 2.63	26.79 ± 2.42	27.16 ± 2.85	0.314
Free T4 (pmol/L)	10.72 (9.95, 11.68)	10.85 (9.96, 11.85)	10.64 (9.83, 11.54)	0.476
Free T3 (pmol/L)	3.71 ± 0.40	3.68 ± 0.41	3.73 ± 0.40	0.380
Maternal TSH (mIU/L)	1.59 (1.10, 2.07)	1.60 (1.14, 2.11)	1.54 (1.09, 2.07)	0.667
*Newborn*				
Birthweight (g)	3432 ± 387	3489 ± 417	3368 ± 341	**0.024**
Cord blood FT4 (pmol/L)	12.77 ± 1.15	12.63 ± 1.28	12.93 ± 0.95	0.059
Cord blood FT3 (pmol/L)	2.41 (2.23, 2.68)	2.42 (2.21, 2.65)	2.40 (2.24, 2.69)	0.847
Cord blood TSH (mIU/L)	4.32 (3.44, 5.25)	4.40 (3.35, 5.20)	4.32 (3.47, 5.47)	0.810

Data are presented as median (interquartile range) or mean ± SD

**Fig. 1:**
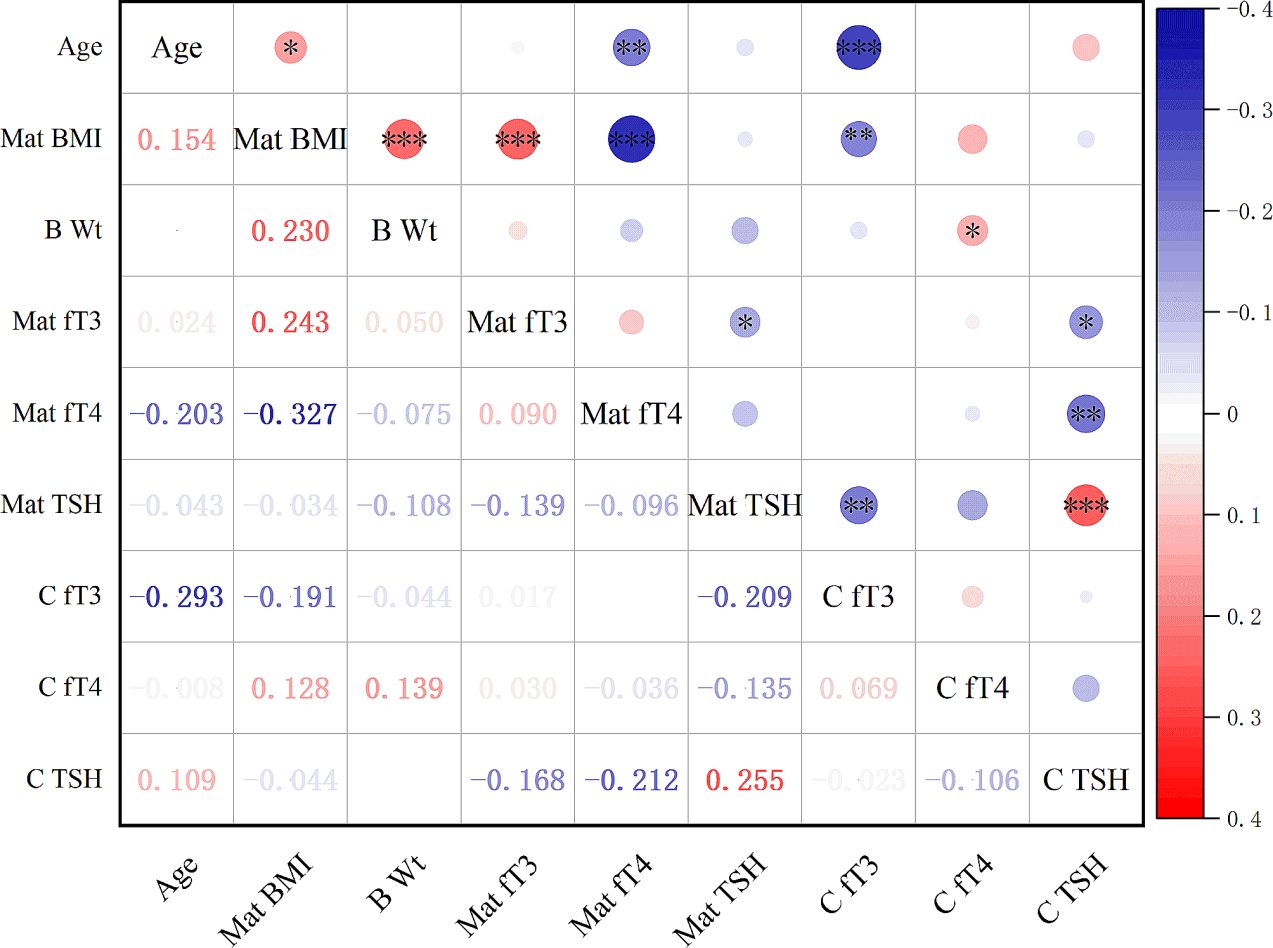
Correlation matrix showing Spearman’s coefficients of bivariate analysis. * *P* < 0.05, ** *P* < 0.01, *** *P* < 0.001. B, birth; C, cord; fT3, free T3; fT4, free T4; Mat, Maternal

The results of bivariate correlation analysis are shown in Fig. 1. The levels of fT3 in cord blood were negatively correlated with maternal age (r_s_ = − 0.293, *P* < 0.001), maternal BMI (r_s_ = − 0.191 *P* = 0.006), and maternal TSH level (r_s_ = − 0.209, *P* = 0.003). fT4 levels showed a weak but significant positive correlation with birthweight (r_s_ = 0.139, *P* = 0.048), but there were no correlations with maternal thyroid parameters. The correlations between TSH and maternal fT3 (r_s_ = − 0.168, *P* = 0.017) and maternal fT4 (r_s_ = – 0.212, *P* = 0.002) levels were negative, while the correlation between cord blood TSH and maternal TSH levels was positive (r_s_ = 0.255, *P* < 0.001).

The association between thyroid hormones in cord blood and maternal characteristics and thyroid hormones in male (*n* = 108) and female (*n* = 95) births was also examined (Table 2). When stratified by offspring sex, the association between maternal age and cord blood fT3 was more negative for female births (r_s_ = − 0.328, *P* = 0.001) than for male births (r_s_ = − 0.270, *P* = 0.005). fT3 levels were inversely associated with maternal BMI in male births (r_s_ = − 0.252, *P* = 0.009) but not in female births. The TSH level of cord blood was inversely associated with maternal fT4 concentration in female births (r_s_ = − 0.233, *P* = 0.023) but not in male births. In female births, the levels were associated only with maternal fT4 (r_s_ = − 0.233, *P* = 0.023). In male births, they were associated with both maternal T3 (r_s_ = − 0.215, *P* = 0.025) and TSH (r_s_ = 0.312, *P* = 0.001) levels.

**Table 2:** Spearman’s correlation between maternal characteristics and neonatal cord thyroid hormones stratified by infant sex

	Cord blood fT3		Cord blood fT4		Cord blood TSH	
	r_s_	*P*	r_s_	*P*	r_s_	*P*
Male birth (*n* = 108)						
Maternal age	**-0.270**	**0.005**	0.056	0.568	0.073	0.450
Maternal BMI	**-0.252**	**0.009**	0.150	0.122	-0.050	0.609
Birthweight	-0.023	0.815	0.168	0.168	-0.012	0.899
Maternal fT3	-0.079	0.417	-0.055	0.575	**-0.215**	**0.025**
Maternal fT4	-0.029	0.763	0.008	0.934	-0.187	0.052
Maternal TSH	-0.145	0.135	-0.098	0.313	**0.312**	**0.001**
Female birth (*n* = 95)						
Maternal age	**-0.328**	**0.001**	0.020	0.847	0.143	0.168
Maternal BMI	-0.119	0.252	0.090	0.385	-0.057	0.580
Birthweight	-0.055	0.593	0.201	0.051	-0.005	0.959
Maternal fT3	0.109	0.293	0.115	0.268	-0.116	0.261
Maternal fT4	0.032	0.761	-0.056	0.591	**-0.233**	**0.023**
Maternal TSH	**-0.282**	**0.006**	-0.178	0.084	0.180	0.081

The variables independently associated with fT3 levels in cord blood were identified via multivariate linear regression analysis, with male and female births analyzed separately. As shown in Table 3, maternal age (standardized β = − 0.274, *P* < 0.001) and the TSH level (standardized β = − 0.212, *P* = 0.002) remained independently and negatively associated with the cord blood levels of fT3 after adjusting for BMI and birthweight in all births. When separated by fetal sex, maternal age was still independently associated with cord blood levels of fT3 in both groups (standardized β = − 0.366, *P* < 0.001 for female births, standardized β = − 0.201, *P* = 0.042 for male births). Maternal TSH values were independently associated with cord blood fT3 in female births (standardized β = − 0.208, *P* = 0.033) but not male births. BMI at delivery was an independent predictor of cord blood fT3 in male births (standardized β = − 0.208, *P* = 0.037) but not in female births.

**Table 3:** Multiple linear regression analysis of cord blood fT3

Independent variables	All births (*n* = 203)			Male births (*n* = 108)			Female births (*n* = 95)		
	Standardized β	95% CI	*P*-value	Standardized β	95% CI	*P*-value	Standardized β	95% CI	*P*-value
Maternal age	**-0.274**	**(-0.408, -0.139)**	**< 0.001**	**-0.201**	**(-0.394, -0.007)**	**0.042**	**-0.366**	**(-0.560, -0.171)**	**< 0.001**
Maternal BMI	-0.089	(-0.227, 0.049)	0.203	**-0.208**	**(-0.403, -0.013)**	**0.037**	0.025	(-0.174, 0.225)	0.801
Birthweight	-0.032	(-0.168, 0.105)	0.648	0.027	(-0.171, 0.225)	0.786	-0.091	(-0.289, 0.108)	0.365
Maternal TSH	**-0.212**	**(-0.346, -0.079)**	**0.002**	-0.185	(-0.380, 0.010)	0.063	**-0.208**	**(-0.399, -0.017)**	**0.033**

## Discussion

We evaluated the association between thyroid hormone levels in cord blood and maternal perinatal factors in euthyroid pregnant women. Free T3 levels were negatively associated with maternal age even after adjusting for confounding factors, and were more robust in female than in male births.

Free T3 is the physiologically active form of thyroid hormone and it regulates metabolism, growth, and development. Recent studies have shown that serum T3 levels can accurately predict tissue T3 content [15]. There is an association between low fT3 and insulin resistance in non-diabetic individuals [16]. Low levels are independent predictors of higher cardiovascular mortality in the general population [17]. Otherwise, it is associated with neurodevelopment during infancy and childhood. For example, higher levels at birth are correlated with optimal performance in personal-social domains during infancy in boys [12], as well as higher normal intelligence [18]. Our findings are consistent with those of a previous study that showed a negative association between maternal age and cord levels of fT3 in euthyroid pregnancy [19]. Taken together, these results suggest that dynamic follow-up of thyroid hormone levels in offspring may shed light on offspring health issues caused by advanced maternal age.

The inverse association between maternal age and cord fT3 levels might be explained by the effects of deiodinases (DIOs), which are abundantly expressed in the placenta and several fetal tissues. In normal pregnancies, thyroid hormone transport from maternal blood to the fetus is regulated by DIO2 and DIO3. DIO2 produces T3 from T4, increasing the availability of T3. DIO3 catalyzes the inactivation of T4 to rT3 and of T3 to diiodothyronine (T2), decreasing the levels of T4 and T3. [20, 21]. Changes in the expression and activity of DIOs, manifesting as a decrease in DIO2 and an increase in DIO3, under hypoxia and oxidative stress have been described. Gutierrez-Vega et al. [14] reported increased DIO3 but decreased DIO2 expression and activity in the placenta in patients with gestational diabetes mellitus, along with a reduced T3 level in cord blood. Furthermore, an association between advanced maternal age and oxidative stress in the placenta has been demonstrated in human pregnancies and in animal experiments. It therefore seems plausible that altered placental DIO expression is linked to the reduced cord fT3 level in older mothers.

During pregnancy, fetal thyroid hormones are influenced by maternal thyroid status. With the increasing development of the thyroid gland and hypothalamic-pituitary-thyroid axis in the fetus during late pregnancy, the dependence on maternal thyroid function decreases. Limited data are available on the relationship between maternal thyroid function in the third trimester and fetal thyroid hormone levels. We found a significantly negative association between cord TSH and maternal fT3 and fT4 levels, as well as a positive association between cord TSH and maternal TSH levels. Velasco et al. [22] also reported this latter association, although another study found no such association between (nor between maternal TSH cord T4 levels) [13]. These discrepancies may be partly due to perinatal factors, including gestational age, delivery mode, and ethnicity but also to differences in measurement methods. Furthermore, it is difficult to explain the differences in these associations between male vs. female births. However, the fetal thyroid system can be disrupted by environmental pollutants, with sex-specific differences [23].

To the best of our knowledge, our study is the first to demonstrate a sex-based disparity in the association between maternal age and neonatal thyroid hormone levels. Specifically, fT3 levels in female fetuses were more sensitive to maternal age. Several studies have suggested that thyroid homeostasis is more vulnerable to disruption in females than in males, but the underlying mechanism is unclear. In animal models, advanced maternal age has a sex-dependent effect on the placental phenotype [24]. We speculate that the observed sexual dimorphism is related to the effects of placental DIO3 but this remains to be confirmed in further studies.

The limitations of our study should also be noted. First, as a cross-sectional study, causality could not be established, such that, despite their correlation, it cannot be concluded that maternal age influences fetal thyroid function. In addition, only healthy pregnant women who underwent scheduled cesarean section were included in this study to avoid potential confounding variables related to delivery and fetal distress, which might affect neonatal thyroid hormone levels. Accordingly, our results cannot be extrapolated to women with complicated pregnancies. Further studies with a more inclusive cohort of pregnant women are therefore needed. Third, data on maternal iodine intake, which might be correlated with maternal and fetal thyroid hormone levels, were unavailable.

## Conclusions

We found a negative association between maternal age and cord blood levels of fT3 in euthyroid pregnant women, with a slightly stronger association for female fetuses. Our findings suggest a potential impact of advanced maternal age on the lifelong health risk of offspring.

## Data Availability

The data that support the findings of this study are available from the corresponding author, upon reasonable request.

## Abbreviations

BMI: Body mass index
DIO: Deiodinase
fT3: Free triiodothyronine
fT4: Free thyroxine
T2: Diiodothyronine
TSH: Thyroid-stimulating hormone
